# DNA Methylation Landscape of ReNcell Common Neural Progenitor Cell Lines Reveals Distinct Lineage Bias

**DOI:** 10.3390/biology15030231

**Published:** 2026-01-26

**Authors:** Martina Gyimesi, Duy L. B. Nguyen, Ian William Peall, Rachel Katherine Okolicsanyi, Larisa Margaret Haupt

**Affiliations:** 1 Centre for Genomics and Personalised Health, Genomics Research Centre, School of Biomedical Sciences, Queensland University of Technology (QUT), 60 Musk Ave., Brisbane 4059, Australia; martina.gyimesi@hdr.qut.edu.au (M.G.); lebaoduy.nguyen@hdr.qut.edu.au (D.L.B.N.); r.okolicsanyi@qut.edu.au (R.K.O.); 2 Stem Cell and Neurogenesis Group, Genomics Research Centre, Queensland University of Technology (QUT), 60 Musk Avenue, Brisbane 4059, Australia; 3 Max Planck Queensland Centre for the Materials Science of Extracellular Matrices, Brisbane 4059, Australia; 4 ARC Training Centre for Cell and Tissue Engineering Technologies, Queensland University of Technology (QUT), Brisbane 4059, Australia

**Keywords:** neural progenitor cells, DNA methylation, lineage specification, Notch signaling, neurogenesis

## Abstract

Neural progenitor cell (NPC) fate decisions are governed by transcriptional and signaling programmes, yet the epigenetic mechanisms stabilising early neuronal versus glial lineage trajectories remain unresolved. Here, DNA methylation landscapes in two widely used human NPC models—ReNcell VM (RVM) and ReNcell CX (RCX)—were examined under several different culture conditions to define regulatory pathways shaping lineage specification. Exploratory analyses revealed that the ReNcell lines exhibited methylation similar to primary glial populations rather than neuronal subtypes, with RCX cells positioned further along a maturation trajectory and RVM cells retaining a multipotent state. RCX cultures displayed hypomethylation of neuronal markers (*DCX*, *ENO2*, *MAP2*), whereas RVM cultures showed consistent *GFAP* hypomethylation, indicative of glial or early progenitor identity. Signaling pathways regulating lineage commitment were highlighted, including TGFβ, Wnt, and Notch signaling. Within the Notch pathway, RCX cells exhibited higher gene expression of *NOTCH2* and *JAG* ligands, consistent with active lateral induction and a developmentally advanced state. In contrast, RVM cells exhibited higher *DLL1* and *NOTCH1* expression, supporting lateral inhibition and cellular heterogeneity. Knockdown of syndecan-4 (*SDC4*) revealed opposing effects on Notch activity. Together, these findings established DNA methylation as a determinant of lineage-specific signaling in human NPCs.

## Simple Summary

Neural progenitor cells are early brain cells that can develop into nerve cells or support cells called glia. These cells are widely used in research to study human brain development and disease, yet it is often assumed that they are neutral starting points that can be directed equally toward different cell fates. In this study, we asked whether two commonly used human neural progenitor cell models already show built-in differences that influence how they develop. We found that both models showed chemical marks on their DNA that were more similar to glial cells than to nerve cells. Cells derived from the cerebral cortex appeared more developmentally advanced, while cells derived from the midbrain retained a more flexible, progenitor-like state. These differences were reflected in epigenetic changes at genes linked to nerve or glial identity and in signaling systems that control cell fate decisions. We also found that altering a cell-surface molecule involved in organising signaling proteins affected these pathways differently depending on the cell model. Together, these findings show that commonly used neural progenitor cells have inherent developmental biases that can be leveraged, rather than resisted, when modelling human brain development and neurological disease, improving experimental design and interpretation.

## 1. Introduction

**Figure 1:**
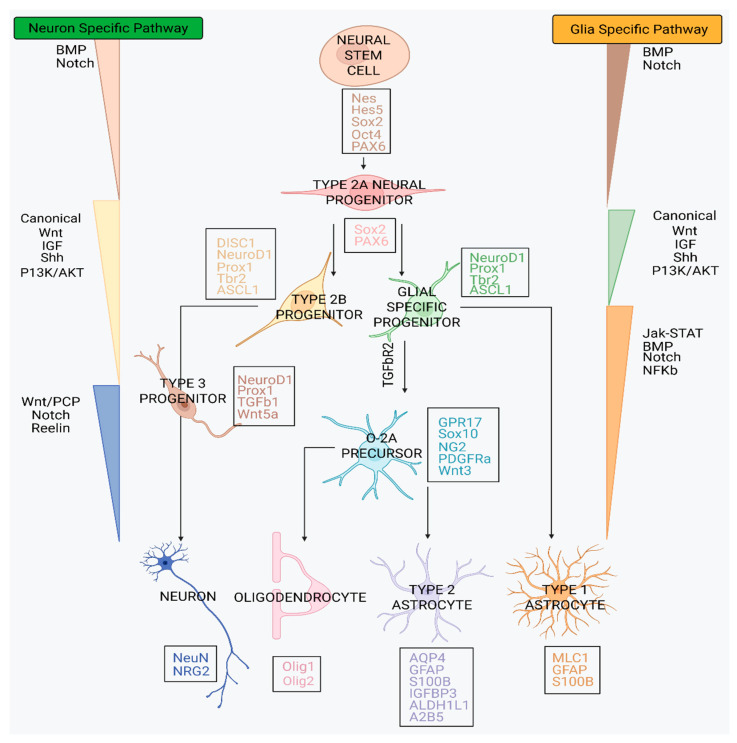
**Neurogenesis and associated gene expression and signaling pathways.** Neurogenesis progresses from neural stem cells (NSCs), which express common stemness and multipotency markers [26], to type 2A neural progenitors marked by decreased expression of self-renewal genes [27]. This transition represents a critical decision point between glial and neuronal lineages [28]. Glial-specific progenitors give rise to type 1 astrocytes—predominantly found in the optic nerve—while oligodendrocytes and type 2 astrocytes emerge from more oligodendrocyte–type-2 astrocyte (O2-A) progenitor types [29,30]. Characteristic gene and protein markers for each stage are presented in boxes along the central developmental pathway colour coded to their relevant cell type. Common signaling pathways that influence lineage specification are illustrated on either side. Triangular shapes denote the relative influence of each signaling pathway: wider bases indicate stronger involvement, whereas narrower tips suggest weaker activity.

Neurogenesis is the process through which neural progenitor cells (NPCs) generate specialised neural cell types and is essential for brain development, neural network plasticity, and tissue repair throughout life [1]. Neurogenesis is governed by tightly regulated transcriptional and signaling programmes that direct lineage commitment of neural stem cells (NSCs) toward neuronal or glial fates [2]. A critical branching point occurs at the Type 2A progenitor stage, where cells transition from self-renewing states to lineage-restricted precursors (Figure 1). Although coordination between Notch-dependent proliferative signals and Wnt-mediated differentiation cues is hypothesised to drive this transition [3,4], the molecular mechanisms that stabilise lineage specification remain incompletely defined.

Disruption of this regulatory balance alters the proportions of progenitors, neurons, and glial cells, and has been implicated in neurodevelopmental abnormalities as well as neurodegenerative disease [5,6]. In Alzheimer’s disease (AD), reduced neurogenic output occurs early in pathology, yet it remains unclear whether this reflects impaired lineage specification, diminished neuronal differentiation, or shifts in the relative composition of progenitor and mature neural populations within neurogenic niches.

Epigenetic regulation, particularly DNA methylation, is central to the NPC fate decision process [7]. DNA methylation modulates transcriptional competence in a stable yet environmentally responsive manner [8,9]. Importantly, methylation dynamics act as an interface between extrinsic factors—including those associated with AD risk—and intrinsic lineage programmes [10]. Despite its recognised importance, the methylation landscape that distinguishes early neuronal versus glial trajectories in human NPCs remain poorly mapped.

Among the modulators of lineage-specifying signaling pathways are the heparan sulfate proteoglycans (HSPGs) [11,12,13]. The sulfation patterns of HSPG glycosaminoglycan chains are established by sequential sulfotransferase activity [14] and confer selective affinity for a wide range of developmental ligands, including fibroblast growth factors (FGFs), Wnt ligands, and Notch receptors [15,16,17]. Through these interactions, HSPGs have the capacity to modulate the crucial Notch–Wnt signaling balance that governs NPC maintenance, proliferation, and lineage progression [18,19]. However, how DNA methylation influences these signaling pathways and their HSPG regulation during early human NPC lineage commitment stages has not been fully examined.

Ethical and practical limitations in studying human neurogenesis *in vivo* have driven reliance on *in vitro* NPC models. While primary human NPCs are constrained by limited expansion potential and variable differentiation efficiency, immortalised ReNcell lines offer reproducibility and accessibility for mechanistic studies [20]. The human NPC lines ReNcell CX (RCX) and ReNcell VM (RVM) have been widely employed as *in vitro* models for the investigation of neurogenesis along with the molecular pathways governing neuronal and glial lineage specification [21,22]. Importantly, although both models are foetal in origin and share a common NPC lineage [23], they differ fundamentally in their regional derivation and intrinsic developmental properties, making them well suited for detailed explorations of inherent molecular differences biasing NPCs toward distinct lineages.

RCX cells were derived from the human foetal frontal cortex, with RVM cells originating from the ventral mesencephalon [23]. In addition to these anatomical differences, the clonally derived RCX cells comprise a relatively homogeneous population with restricted lineage potential. In contrast, RVM cells, generated from bulk tissue isolation, yield a more heterogeneous population, retaining broader developmental plasticity [23]. Comparative investigations of RCX and RVM cells provide a unique experimental framework to examine how regional identity and intrinsic progenitor properties shape molecular regulation during early neurogenesis [24,25]. Crucially, the close alignment of these models in terms of developmental stage (foetal NPC origin) enables the capacity to inform a meaningful contrast between cortical and mesencephalic neurogenic programmes. As such, parallel analyses of RCX and RVM cells offer a valuable insight into region-specific regulatory mechanisms that may contribute to divergent neuronal outcomes along with differential vulnerability in neurodevelopmental and neurodegenerative contexts.

Here, we investigated how DNA methylation landscapes differ between RCX and RVM models across basal, neuro-inductive (SD), heparin-modulated, and neurosphere (IN) conditions. The integration of environmental perturbation (added heparin and distinct culture conditions) with genome-wide methylation profiling (Illumina EPIC array) was used to identify epigenetic signatures associated with neuronal versus glial lineage trajectories and to determine how HSPG-mediated signaling may contribute to early human NPC fate specification.

## 2. Materials and Methods

### 2.1. ReNcell Cell Models

ReNcell CX (SCC007) and ReNcell VM (SCC008) human NPC lines were procured from Merck Millipore (Sigma-Aldrich, Burlington, MA, USA). The ReNcell CX model was clonally derived from the frontal cortex of a 14-week gestational age human foetus [23] and immortalised with c-myc oncogen. In contrast, the ReNcell VM model was established from bulk tissue isolation of the ventral mesencephalon from a 10-week gestational age human foetus [23] and immortalised using the v-myc oncogene. Both cell lines were isolated in a legal and ethical manner compliant with local informed consent procedures, with further information found in Donato et al. [23].

### 2.2. ReNcell Cell Culture

GelTrex™ (15 mg/mL; Thermo Fisher Scientific, Scoresby, VIC, Australia, Cat# A1413202) and laminin (1 mg/mL; Invitrogen, Carlsbad, CA, USA, Cat# 23017-015) were defrosted overnight at 4 °C. Attachment factors were diluted to working concentrations of 0.1 mg/mL (GelTrex™) or 20 µg/mL (laminin) in cold ReNcell growth media and 1X phosphate-buffered saline (PBS; Gibco™ Scoresby, VIC, Australia, Cat# 4190144), respectively. ReNcell growth media contained Dulbecco’s Modified Eagle Medium (DMEM), F-12, GlutaMAX-I (Cat#10565018, Gibco™) 2% B-27 supplement (Cat#17504001, Gibco™), and 1% Penicillin-Streptomycin (Cat#15140122; Thermo Fisher Scientific). The diluted solutions were added to p100 (Corning^®^, Mulgrave, VIC, Australia, Cat#CLS4615) flasks and incubated at 37 °C for 2 h (GelTrex™) or 4 h (laminin) before being removed. ReNcell CX and VM cells (1 × 10^6^) were thawed and resuspended in 2 mL of pre-warmed growth media, then centrifuged at 300 rcf for 5 min. Freeze media, containing 20% dimethylsulfoxide (DMSO; Invitrogen™, Cat#D12345) with 80% ReNcell growth media, was removed prior to resuspending the cells in 10 mL of fresh, pre-warmed ReNcell growth media supplemented with 20 ng/mL fibroblast growth factor 2 (FGFb; Cat# PHG0026, Thermo Fisher Scientific) and 20 ng/mL epidermal growth factor (EGF; Cat# PHG0311, Thermo Fisher Scientific). Cell cultures were incubated at 37 °C with 5% CO_2_. ReNcell growth media was exchanged the following day and subsequently every other day. Cells were allowed to reach approximately 80% confluency before being passaged using 5 mL of TrypLE (Cat#12604013, Gibco™). Cultures were incubated for 5 min at 37 °C and 5% CO_2_ to facilitate dissociation, after which cells were gently resuspended. Following centrifugation at 300 rcf for 5 min, cells were resuspended in fresh, pre-warmed ReNcell growth media and plated onto pre-coated experimental plates. Seeding densities were set at 2500 cells/cm^2^ for dosing experiments and 15,000 cells/cm^2^ for neuroinductive assays. For heparin dosing, porcine heparin salt was diluted in PBS (1 mg/mL; Cat#H3149-100KU, Sigma-Aldrich) and was added to cultures at final concentrations of 1 µg/mL and 10 µg/mL. Cultures were maintained for 7 days with RNA harvested at Days 3, 5, 7 and gDNA harvested at Day 7. Basal ReNcell cultures were abbreviated to RCX (ReNcell CXs) and RVM (ReNcell VMs) with respective heparin concentrations marked by 0, 1, or 10.

### 2.3. Induced Neurosphere Cell Culture

ReNcell cell lines were defrosted in a 37 °C water bath until completely thawed, then transferred to a 50 mL Falcon tube containing 9 mL of ReNcell growth media. Following centrifugation at 300 rcf for 5 min, and aspiration of the supernatant, cells were resuspended in ReNcell growth media supplemented with freshly added FGFb and EGF (at final concentration of 20 ng/mL) and plated onto Geltrex™-coated p100 culture plates. To generate induced neurospheres (INs), cells were dissociated using TrypLE (as above) and neutralised with an equal volume of ReNcell growth medium. Cells were then pelleted (300 rcf for 5 min), resuspended in fresh ReNcell growth media containing FGFb and EGF, and plated in biological triplicate into low-attachment 24-well plates (Cat#CLS3474; Corning^®^ Costar^®^, Mulgrave, VIC, Australia). INs were maintained for 7 days, with half ReNcell media changes (aspirating only 250 µL from a 24-well plate) and resupplementation of FGFb, EGF, and heparin (0, 1, and 10 µg/mL) performed on Days 2, 4, and 6. RNA and gDNA were harvested in biological triplicates at the end of the culture period and described in more detail below. ReNcell INs were abbreviated to RCX IN (ReNcell CX neurospheres) or RVM IN (ReNcell VM neurospheres) with respective heparin concentrations marked by 0, 1, or 10.

### 2.4. Neuro-Inductive Cultures

For neuro-inductive cultures of ReNcell models, FGFb and EGF were removed from the ReNcell growth medium, and cells were maintained for 14 days, with full media changes (and heparin resupplementation) performed every other day. RNA and gDNA were extracted on Day 14. Neuroinducted cultures were abbreviated to RCX SD (ReNcell CX spontaneously differentiated cultures) or RVM SD (ReNcell VM spontaneously differentiated cultures) with respective heparin concentrations marked by 0, 1, or 10. Neuroinduction of IN cultures began with IN formation as previously described, followed by transfer of INs onto Geltrex™—or laminin-coated 24-well plates. From that point, FGFb and EGF were omitted for an additional 14 days, resulting in a total culture duration of 21 days. Due to their fragility and smaller size, ReNcell CX INs were induced in low-attachment p100 dishes (Cat#CLS4615; Corning^®^) before being transferred to 6-well plates (Cat#140675; Corning^®^) to allow collection of a greater volume of material for RNA and gDNA. These cultures were abbreviated to RCX IN SD (ReNcell CX neurospheres spontaneously differentiated) or RVM IN SD (ReNcell VM neurospheres spontaneously differentiated) with respective heparin concentrations marked by 0, 1, or 10.

### 2.5. siRNA Knockdowns

Accell siRNA SMARTpool reagents (Cat# DHA-B-005000-500; Dharmacon™, Lafayette, CO, USA) were used to knock down (KD) expression of *SDC4* (Cat# E-003706-00-0005; Dharmacon™) in ReNcell cell cultures. Both ReNcell lines were seeded at 2500 cells/cm^2^ and cultured to 60–70% confluency. Cells were then changed to reduced B-27 supplement (0.4%) to synchronise cell cycles for 24 h prior to transfection with Accell siRNA (Dharmacon™) at a working concentration of 1 µM. Cells were subsequently changed to Accell siRNA delivery media. Each experiment was performed in biological triplicates. Sample groups included untreated cells (UT) including only delivery media, negative control siRNA (Accell non-specific targeting siRNA; scramble (SCR); Cat#D-001910-01-05; Dharmacon™), and KD cultures with relevant siRNA added. Cells were incubated at 37 °C in 5% CO_2_ for 72 h, without media change, before RNA and gDNA were collected. For downstream analysis, UT and SCR were averaged to provide a control for KD gene expression analysis.

### 2.6. RNA Extraction, cDNA Synthesis, QPCR

RNA extraction was conducted using TRIzol^®^ reagent (Invitrogen) and the Direct-zol™ RNA miniprep kit (Zymo Research, Irvine, CA, USA, Cat# R2050) following manufacturer’s protocols, which included in-column DNase I treatment (Zymo Research). Subsequent cDNA synthesis utilised 150 ng of purified RNA and the iScript cDNA synthesis kit (Bio-Rad Laboratories, Gladesville, NSW, Australia, Cat# 170-8891). Quantitative PCR (Q-PCR) reactions were performed in quadruplicate for each gene of interest within a 384-well microtiter plate (Thermo Fisher Scientific). This meant each biological triplicate harvested was added in quadruplicates to the QPCR plate, resulting in 12 technical replicates for each gene of interest. Each reaction comprised 120 ng of cDNA template, 5 μL of 1X SYBR^®^-Green PCR Master Mix (Promega, Alexandria, NSW, Australia, Cat# A6001), 200 ng of forward and reverse primers (Integrated DNA Technologies, Coralville, IA, USA), and 0.1 μL of CXR reference dye (Promega). Amplification progress was monitored using a Life Technologies QuantStudio™-7 (Thermo Fisher Scientific). Cycle threshold (Ct) values were normalised against the Ct values of the endogenous control gene 18S (ΔCt) included in each run as previously [11]. Gene expression levels were determined by calculating the ΔΔCt value (2^(−ΔCt)^), and relative gene expression values were scaled by 1 × 10^6^ for easier visualisation as bar graphs or to present as a single number with standard deviation (2^(−ΔCt)^ × 10^6^ ± standard deviation). To analyse any previously unpublished gene expression data, relative gene expression values were subjected to two-way ANOVA with multiple comparisons as a statistical test.

### 2.7. DNA Methylation Array

Genomic DNA methylation levels were assayed using Illumina Infinium (EPIC 850K) arrays as previously described [31]. Biological triplicates were pooled into a single replicate within the Illumina EPIC beadchip. Analyses were performed using R (version 4.2.0), adapting previously established workflows incorporating functional normalisation prior to data exploration [13,32,33]. Methylation was annotated by IlluminaMethylationEPICanno and Manifest packages (GRCh37.p13). CpG sites harbouring common single nucleotide polymorphisms (SNPs; minor allele frequency of >0.1) were excluded. Pairwise comparisons of beta values (proportion of methylated to unmethylated cytosines) identified absolute differences in cell models at individual CpG sites. Beta values ranged from 0 to 1, with 0 representing hypomethylation (0% methylation) and 1 representing hypermethylation (100% methylation). Beta values were considered as percentage (0.4 = 40%) of cells methylated at the CpG site. Percentage differences in CpG sites were noted above 10%, with a difference above 20% considered significant.

### 2.8. Statistical Analysis

DNA methylation beta values were extracted from the normalised and filtered EPIC array data stored in a *QuantRatioSet* object. Principal component analysis (PCA) was performed on selected samples and differential methylation was assessed using the *mCSEA* version 1.10.0 (methylation-based Gene Set Enrichment Analysis) method, implemented with the *mCSEATest* function. Changes in beta values between cell conditions were calculated, and the rank-based *mCSEA* was run with a minimum CpG set size of 5, utilising parallel processing and the EPIC platform annotation. This resulted in *p*-values which were then adjusted to multiple testing. To assess global DNA methylation differences, the genome was divided into 1 million basepair (bp) bins. This binning approach reduced the impact of site-specific variation and provided an overall view of methylation differences across all samples. Beta values were averaged for each sample within these regions to easily distinguish global methylation over CpG site-specific differences. Correlation analyses between samples and features were performed using Pearson’s correlation coefficients. Heatmaps were generated to visualise methylation patterns and sample clustering, employing the *pheatmap* (version 1.0.13) R package, with hierarchical clustering applied to both samples and CpG sites to explore relationships and groupings within the data. Protein–protein interaction networks were constructed using the STRING database (www.string-db.org, accessed on 21 March 2024) to explore functional associations between the top genes mapped to differentially methylated CpG sites and to identify key hubs potentially involved in the underlying biological mechanisms.

### 2.9. Gene Expression Omnibus (GEO) Data Retrieval and Analysis

Publicly available Illumina EPIC 850K methylation array datasets were retrieved from the NCBI Gene Expression Omnibus (GEO) database (https://www.ncbi.nlm.nih.gov/geo/ accessed on 6 February 2025). Relevant datasets were identified using specific search terms related to the study focus (e.g., neural lineages, DNA methylation). Raw and processed data files were downloaded from the GSE234520 (previously published [34]) dataset and preprocessed together with ReNcell data as above, including quality control, normalisation, and differential methylation analysis. GSE234520 is a DNA methylation dataset generated using the Illumina EPIC (850K) array on sorted nuclei from human cortical tissue. Samples were enriched for distinct brain cell types, including neurons, oligodendrocytes, astrocytes, and microglia, providing reference methylation signatures to deconvolute bulk brain data and investigate cell type-specific epigenetic variation. Beta values from GSE234520 were averaged across sample replicates (neuron *n* = 28; astrocyte *n* = 7; glial *n* = 21) to generate a single representative value per CpG site [34].

## 3. Results

### 3.1. ReNcell Methylation Profiles Reveal Glial Lineage Similarity

**Figure 2:**
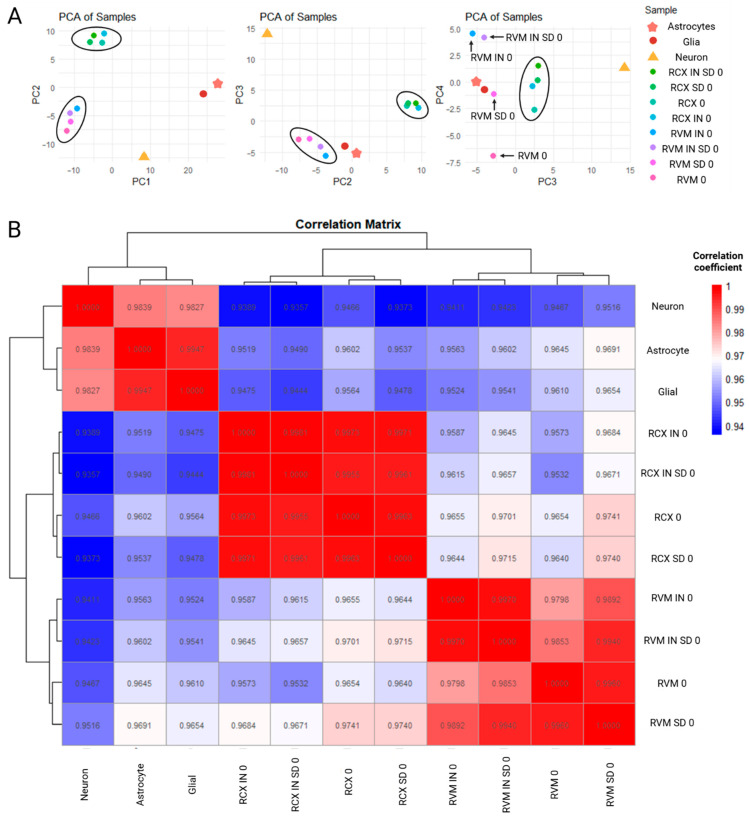
**Principal component analysis (PCA) and correlation analysis of ReNcell and primary neural populations.** (**A**) PCA of ReNcell CX (RCX), ReNcell VM (RVM), glial (astrocytic + oligodendrocytic), astrocytic-only, and neuronal populations (GSE234520, [34]). ReNcell VM cultures showed closer proximity to glial/astrocytic populations along PC2 and PC3, whereas PC4 indicated that neuroinducted RVM monolayers (RVM SD 0) were most similar to glial samples. RVM neurospheres (RVM IN 0, RVM IN SD 0) formed a distinct cluster, while RCX populations remained tightly grouped across components. (**B**) Pearson correlation matrix (r = 0.9–1.0) comparing CpG methylation profiles across ReNcell progenitors, mature astrocytes, neurons, and glial populations. Both ReNcell lines showed strongest correlations with glial/astrocytic groups, with RCX 0 and RVM 0 cultures also displaying moderate similarity to neuronal populations. Neuroinducted RVM cultures (RVM IN SD 0 and RVM SD 0) exhibited an unexpected increase in correlation with RCX 0, suggesting divergence in neuro/gliogenic trajectories.

Beta values were extracted from RCX 0 and RVM 0 culture samples and compared to mature neural lineages from post-mortem brain samples (extracted from GSE234520), with a focus on grouped glial, astrocyte, and neuronal lineages comparisons. Major differences among RCX 0, RVM 0, Neuronal, Glial, and Astrocytic samples were revealed by principal component analysis (PCA) along PC1 and PC2 (Figure 2). While the CpG sites driving PC1 (distinguishing cell types) were mapped to genes primarily involved in cell cycle and ion transport (*POLA1* and *SLC9A1*), CpG sites driving PC2 positively (distinguishing all RCXs from all RVMs) mapped to developmental and neuronal transcription factors such as *FOXA1*, *FOXB1*, *FOXP2* with genes like *GATA2*, *PITX1*, and *DLX1* driving it negatively. However, when transposed to PC3, a greater similarity to glial and astrocytic lineages was exhibited by RVM 0. Further analysis of PC3 and PC4 demonstrated that the highest resemblance to these mature glial populations was displayed by neuroinducted RVM SD 0. This similarity showed mostly on PC4 where RVM 0 were distinguished by a significant negative loading primarily driven by CpG sites within genes involved in membrane transport such as *TMEM175*, *KPNA1,* and *SEC14L1*. The PCA results for each principal component, including the top 20 CpG sites contributing most strongly to each component, are presented in Supplementary Material (Table S1).

To further investigate the relationship between cell models, a correlation matrix was generated using methylation data averaged across 1-million base pair genomic bins. This approach enabled a genome-wide overview of DNA methylation similarities and differences. Pearson correlation coefficients were calculated, with values closer to 1 indicative of greater similarity. Among the samples analysed, RVM SD 0 exhibited the highest similarity with mature neurons (Pearson’s r = 0.9516). RVM SD 0 samples also demonstrated the strongest correlation with astrocyte profiles (Pearson’s r = 0.9601). Interestingly, RVM IN SD 0 were identified to display a lower correlation with the astrocyte lineage (Pearson’s r = 0.9602) when compared with RVM 0 cultures (Pearson’s r = 0.9645).

While all RCXs samples were generally shown to have lower similarity to astrocyte and glial profiles when compared to all their RVM counterparts, RCX 0 cultures were found to exhibit the highest correlation to mature neuronal lineages (Pearson’s r = 0.9466). Notably, neuroinduction and IN formation reduced these correlations, indicating a shift in the methylation pattern of the cultures away from those observed in mature neural populations. Remarkably, both RCX and RVM populations were found to be as dissimilar from one another as they were from mature neural cell types. The strongest inter-cell line correlation was observed between basal RCX 0 and RVM SD 0 (Pearson’s r = 0.9741), and between RCX SD 0 and RVM SD 0 cultures (Pearson’s r = 0.9740). Conversely, the lowest correlation in terms of the lineage methylation profile was identified between RCX IN SD 0 and RVM 0 cultures (Pearson’s r = 0.9532).

**Figure 3:**
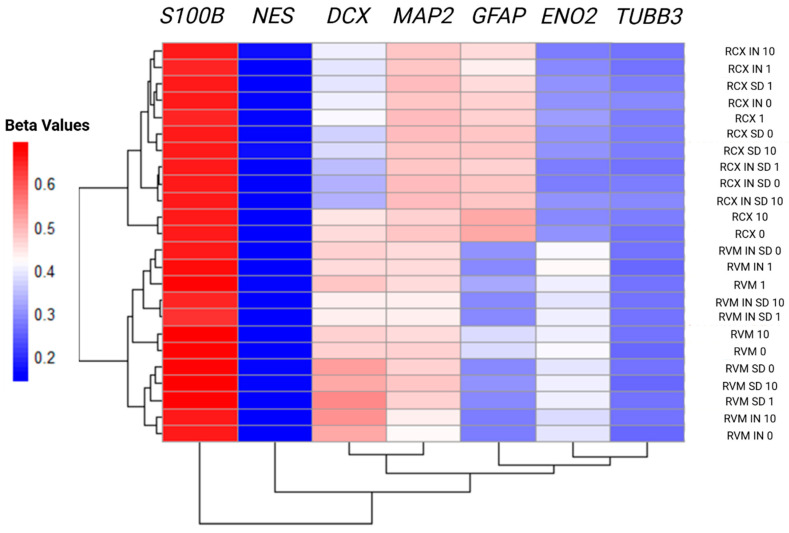
**Hierarchical clustering of methylation patterns in neural lineage markers.** Panels display beta values representing DNA methylation levels, with blue indicating low methylation (potentially permissive for gene expression) and red indicating high methylation (potentially repressive). Each value reflects the average methylation across all CpG sites associated with a given gene. For reference, a beta value of 0.3 suggests ~30% methylation at that locus. Abbreviations: *S100B*—S100 calcium-binding protein B; *NES*—Nestin; *DCX*—Doublecortin; *MAP2*—microtubule-associated protein 2; *GFAP*—glial fibrillary acidic protein; *ENO2*—neuron-specific enolase; *TUBB3*—Tubulin beta-3; RCX—ReNcell CX; RVM—ReNcell VM; IN—induced neurosphere; SD—spontaneous differentiation (neuroinductive culture); 0, 1, 10, heparin concentrations (µg/mL).

To further investigate lineage-specific signatures, we assessed the average methylation levels of key neural marker genes, normalised to the number of CpG sites per gene, thereby generating a single representative methylation value per gene per sample. This analysis included heparin-treated samples and focused on markers across neural development stages: Nestin (*NES*; marker of neural stem cells), Tubulin beta-3 (*TUBB3*; marker of immature neuronal progenitors), glial fibrillary acidic protein (*GFAP*) and S100 calcium-binding protein B (*S100B*; markers of glial cells), and doublecortin (*DCX*; markers of immature neurons) enolase 2 (*ENO2*) and microtubule-associated protein 2 (*MAP2*; markers of mature neurons) (Figure 3). Notably, *DCX* methylation patterns revealed that RCX 0 and RCX 10 samples clustered closer to RVM cultures, which showed intermediate methylation levels (beta values ~ 0.4–0.5). All other RCX samples exhibited lower *DCX* methylation (beta value < 0.4), indicative of a more neuronal or mature trajectory than RVMs. Interestingly, 1 µg/mL heparin treatment further reduced *DCX* methylation in RCX 1, resembling the profile of RCX IN and SD cultures, whereas heparin had a minimal effect on *DCX* in ReNcell VMs. In contrast, *GFAP* methylation levels in RVMs were found to be more responsive to 1 µg/mL heparin, which reduced methylation and shifted the cell profile toward that of RVM IN and RVM SD conditions—showcasing the glial trajectory.

### 3.2. Differentially Methylated Genes in ReNcell Models Reveal Distinct Signaling Pathways Governing Each Cell Type

**Figure 4:**
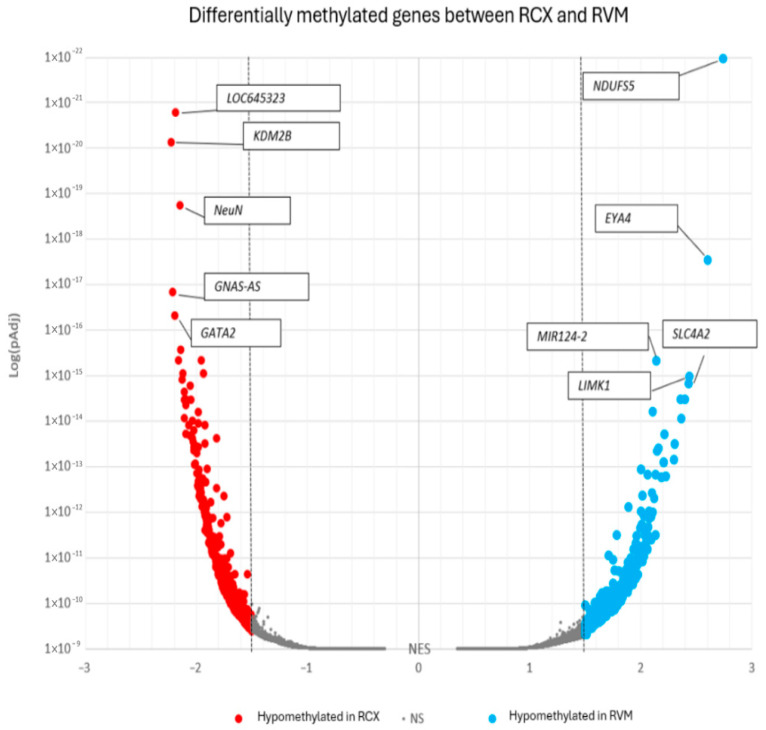
**Volcano plot of differentially methylated promoters between monolayer ReNcell CX (RCX) and VM (RVM) cultures.** This plot was generated using delta beta values from CpG sites comparing RCX and RVM cultures. CpG methylation levels were analysed across genome-wide promoter regions, with statistical significance increasing as larger methylation differences were observed over more CpG sites in gene promoters. Promoters that were hypomethylated in RCX cells but hypermethylated in RVM cells are highlighted in red, while those hypermethylated in RCX cells and hypomethylated in RVM cells are shown in blue. The *x*-axis represents Normalised Enrichment Scores (nES), with values exceeding 1.5 highlighted. The *y*-axis denotes log-transformed *p*-values adjusted for multiple testing. Top gene abbreviations are *MIR9-2HG* (*LOC645323*)—MIR9-2 Host Gene; *KDM2B*—Lysine Demethylase 2B; *NeuN*—Neuronal Nuclei; *GNAS-AS*—GNAS Antisense RNA; *GATA2*—GATA Binding Protein 2, *NDUFS5*—NADH:Ubiquinone Oxidoreductase Subunit S5; *EYA4*—EYA Transcriptional Coactivator and Phosphatase 4; *MIR124-2*—MicroRNA 124-2; *SLC4A2*—Solute Carrier Family 4 Member 2; *LIMK1*—LIM domain kinase 1.

Promoter, gene, and CpG island enrichment analyses were then explored to identify differentially methylated loci between the RCX and RVM models (Figure 4). Particular focus was placed on genes with hypomethylated promoters, which are typically indicative of active or permissive transcription. In RCX cultures, the promoter with the most significant differential methylation, as determined by enrichment score, was identified as *KDM2B* (Normalised Enrichment Score; (nES) = −2.23, adjusted *p*-value; (padj) = 7.39 × 10^−12^), whereas *MIR9-2HG* (also known as *LOC645323*) exhibited the most significant *p*-value (nES = −2.19, padj = 1.63 × 10^−16^). In contrast, the most differentially methylated promoter in RVMs, based on highest nES and lowest padj, was *NDUFS5* (nES = 2.74, padj = 1.08 × 10^−13^). Promoter hypomethylation in RCX cultures was found to be significantly enriched in genes associated with neurogenesis and neuronal differentiation, including *TBR1* (padj = 0.0410), *SATB2* (padj = 0.00031), *NEUROD1* (padj = 0.0319), *SOX9* (padj = 0.00183), *GLI3* (padj = 0.00021), *TGFB2* (padj = 0.00932), *TGFB3* (padj = 0.04196), and *FZD1* (padj = 0.00331). In contrast, a more variable pattern of gene promoter hypomethylation was observed in RVM cultures, primarily affecting histone-related genes. When DNA methylation was examined in regions of lower regulatory activity (e.g., within the body of the gene), hypomethylation was detected in several genes implicated in NPC maintenance and proliferation, including *PROX1* (padj = 0.00690), *NR2F2* (padj = 0.01747), *NKX6-1* (padj = 0.01511), *WNT5B* (padj = 0.01643), and *NRG1* (padj = 1.47 × 10^−6^).

Given the established involvement of these genes in the TGFb and Wnt signaling pathways, methylation differences in pathway-related genes were then examined across the cell models. RCX cultures were found to exhibit consistent hypomethylation across the *TGFB3*, *TGFB1*, and *TGFB2* genes, suggesting these cells to be in a more epigenetically permissive state. Among these, *TGFB3* demonstrated the lowest methylation levels in RCX cultures under RCX IN SD conditions, yet remained responsive to changes in the culture environment (i.e., the addition of heparin), indicating a process of dynamic epigenetic regulation. In contrast, *TGFB2* methylation in RVM cultures was observed to be more variable and appeared more sensitive to changes culture conditions, with the lowest levels detected in SD cultures. This data highlighted cell line-specific difference in the epigenetic regulation of TGFb signaling components between the two models. With respect to the Wnt signaling pathway, prior analyses established *WNT5B* promoters to be distinctly hypomethylated in RVM monolayer cultures. To build upon this finding, methylation of *WNT5A*, another key ligand of the non-canonical Wnt pathway, was also assessed. *WNT5A* was found to be consistently hypomethylated across all RCX culture conditions, suggesting lineage- or cell type-specific epigenetic priming for non-canonical Wnt signaling in these cells. For exploratory heatmaps within these signaling pathways, see Supplementary Material (Figure S1).

### 3.3. Distinct Notch Pathway Methylation in ReNcells

Analysis of the array methylation data identified the top 1000 CpG sites exhibiting significant differential methylation across cellular models. Gene enrichment analysis was subsequently performed using the STRING database (multiple protein analysis, version 12.0), through which differentially enriched gene ontology networks within the cell models were determined. For this analysis, genes with at least two CpG site occurrences in the top 1000 list between the RCX and RVM models were selected. A strength threshold of 1.5 for statistical significance was applied, and the top gene ontology terms enriched between the two monolayer cultures summarised (Table 1).

While most enriched pathways were identified to be associated with lineage specificity and supported previous findings from our group and others, the notable enrichment of the negative regulation of the Notch signaling pathway in RCX models was interpreted as a potential regulatory mechanism that not only underpinned neural lineage specificity but also closely linked different stages of neurogenesis. Additionally, this analysis highlighted the Paired Like Homeodomain 1 (*PITX1*), Zinc Family Member 1 (*ZIC1*), GATA Binding Protein 2 (*GATA2*), F2R Like Trypsin Receptor 1 (*F2RL1*), Siah E3 Ubiquitin Protein Ligase 3 (*SIAH3*), and Sushi, Nidogen and EGF Like Domains 1 (*SNED1*) as key genes exhibiting the most pronounced differences in CpG site beta values and as such level of methylation. Specifically, these genes were found to be completely unmethylated in RCX, with hypermethylation observed in RVM cultures.

### 3.4. Methylation and Expression of Notch Pathway-Specific Genes

**Figure 5:**
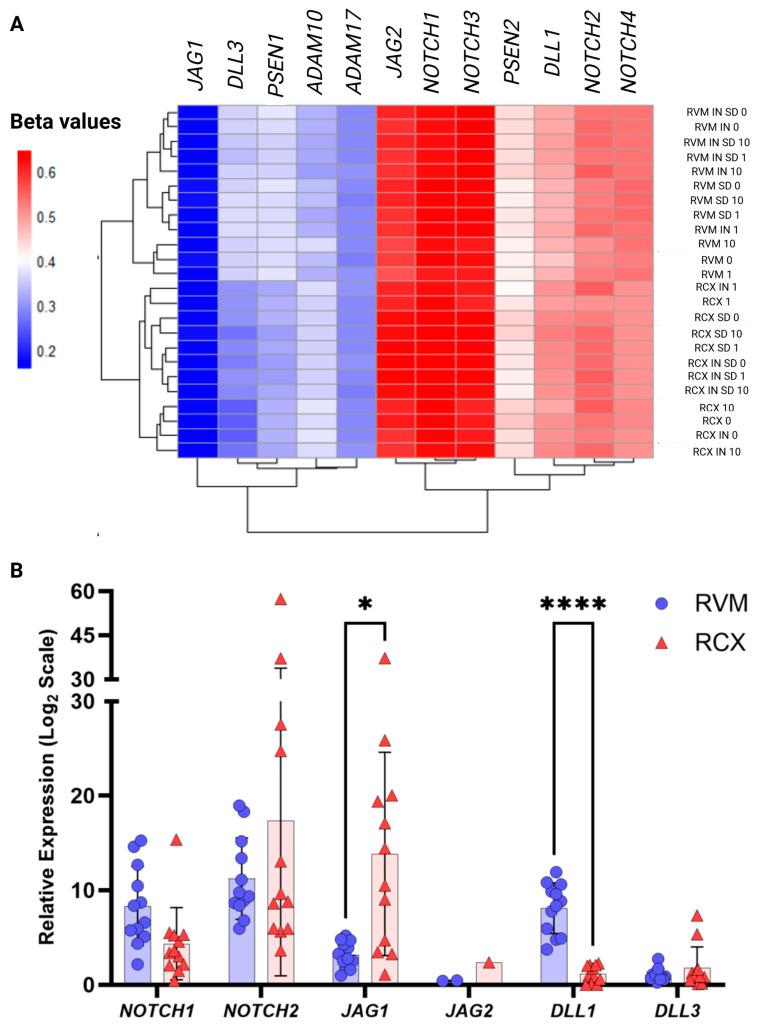
**Methylation and gene expression profiling of Notch pathway components in ReNcell CX (RCX) and VM (RVM) models.** (**A**) Mean beta values were calculated for each gene by averaging methylation levels across all associated CpG sites associated with respective genes on the Illumina EPIC array. Beta values range from 0 (unmethylated) to 1 (fully methylated), visualised on a colour scale from 0.2 (blue; low methylation) to 0.6 (red; higher methylation). Each column represents a Notch pathway gene, and each row corresponds to a RCX or RVM condition (basal, neuroinductive-SD, and neurosphere-IN, ±heparin at concentrations of 0, 1 and 10 µg/mL). Hierarchical clustering was used to group genes with shared methylation patterns. While most Notch-related genes displayed broadly similar methylation profiles across conditions, distinct differential methylation was observed in key regulators including *DLL3*, *PSEN1*, and *ADAM10*, with more subtle variation in *PSEN2*, *DLL1*, and *NOTCH2.* (**B**) Relative gene expression levels were quantified using the ∆∆Ct method and scaled by 10^6^ for visualisation. Statistical significance is indicated by * *p* < 0.05 and **** *p* < 0.0001. *NOTCH1* and *NOTCH2* represent the predominant Notch receptors expressed in these cultures, whereas *JAG1, JAG2*, *DLL1,* and *DLL3* encode the corresponding receptor ligands. Expression differences between RCX and RVM reflect divergent regulation of the Notch pathway consistent with their distinct lineage tendencies and developmental stages.

The methylation patterns of Notch pathway components were then investigated in the RCX and RVM cell models cultured under IN and SD conditions, along with a comparison following heparin addition to the cultures. While most Notch-related genes were found to display only subtle methylation differences between the two cell models, notable differences were detected in the *DLL3*, *PSEN1*, and *ADAM10* genes, with smaller but discernible variations observed in the *PSEN2*, *DLL1*, and *NOTCH2* genes (Figure 5). These differences were found to primarily reflect changes to averaged beta values across the genes, with minimal CpG site-specific variation.

Interestingly, a prominent methylation difference in the *JAG1* gene was identified at the *JAG1*:cg13592599, located within a key transcription starting site (TSS)-associated CpG island (chr20:10650660–10653795), where higher methylation levels were observed in RVM (beta value > 0.5) when compared to RCX cultures (beta value < 0.3). Methylation of the *JAG2* gene was found to be reduced in RVM cultures following heparin treatment (10 µg/mL), particularly at *JAG2*:cg18212924 and *JAG2*:cg14977279 (with 1 µg/mL), where methylation was reduced by approximately 13% and 70%, respectively, with these CpG sites located within the 3′UTR CpG island (chr14:105608159–105626999). Examination of the *DLL1* gene identified methylation to remain largely consistent, with the *DLL3* gene found to demonstrate substantial variability across the models and the different culture conditions. While RVM cells were found to maintain a relatively stable *DLL3* methylation profile, RCX cells exhibited pronounced fluctuations at *DLL3*:cg07640648 and *DLL3*:cg08551532. With both sites positioned on exon boundaries, this suggests a potential regulatory role for this gene in mRNA variation.

In the *PSEN1* gene, CpG-specific differences were observed at the promoter-associated sites *PSEN1*:cg09970967 and *PSEN1*:cg26124115, with lower methylation levels observed in RCX (beta value < 0.5 and 0.2, respectively), and higher levels for these sites observed in RVM cultures (beta value > 0.6 and 0.3, respectively). Conversely, the *PSEN2, NOTCH1*, *NOTCH2*, *ADAM10*, and *ADAM17* genes were identified to display consistent methylation patterns across both cell models and across the various culture conditions explored in this study (Figure 5A).

To evaluate the potential biological relevance of these methylation differences in NPCs, the corresponding gene expression profiles of the genes identified to have differential methylation profiles was then assessed by QPCR (Figure 5B). Notably, *PSEN1* gene expression was found to be significantly elevated in RCX cells (60.38 ± 36.91) when compared to RVM cell cultures (14.89 ± 4.20), correlating with the observed pattern of promoter methylation. Similarly, examination of *ADAM10* and *ADAM17* gene expression identified these genes to be more highly expressed in RCX cells (*ADAM10*: 22.40 ± 3.23; *ADAM17*: 11.67 ± 0.84) when compared to RVM cultures (*ADAM10*: 6.73 ± 0.83; *ADAM17*: 4.58 ± 0.74). Among the Notch receptors, *NOTCH1* and *DLL1* gene expression was identified to be more highly expressed in RVM cultures (*NOTCH1*: 8.33 ± 4.16; *DLL1*: 8.09 ± 2.68), with the *NOTCH2* and *JAG1* found to have stronger gene expression in RCX cultures (*NOTCH2*: 17.35 ± 16.41; *JAG1*: 13.84 ± 10.76). This data suggested a distinct ligand–receptor interaction landscape between the two cell models, potentially relevant to neural lineage specification and the stages of lineage commitment.

### 3.5. Notch Pathway Activation Consistent with Neurogenesis Timeline of ReNcell Models

**Figure 6:**
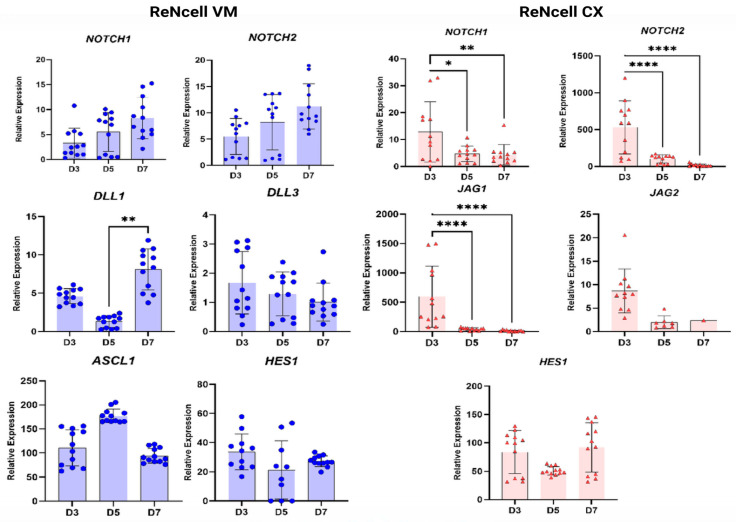
**Relative expression of Notch pathway genes in basal ReNcell VM (Blue-RVM 0) and CX (Red-RCX 0) cultures over a 7-day expansion period**. Relative gene expression levels were calculated using the qPCR ∆∆Ct method multiplied by 10^6^ to enhance visualisation. Statistical significance is indicated by asterisks (* *p* < 0.05; ** *p* < 0.005; **** *p* < 0.0001). These data highlight contrasting dynamics of Notch signaling components between the two cell models during basal expansion.

To explore Notch pathway gene expression in the cell models, ReNcells were then investigated across a 7-day basal expansion culture. Decreased expression of Notch pathway-related genes was observed in RCX, with an increase in expression in these genes demonstrated in RVM (Figure 6) by Day 7 of 2D monolayer cultures. This observation was notable given that activation of the Notch pathway requires direct cell-to-cell contact, which would be expected to increase as cultures became more confluent. Interestingly, gene expression in RCX cultures was found to be highest at Day 3, when the cells were still relatively dispersed within the culture environment, and lower at D7, when confluency was estimated to have reached approximately 80%.

### 3.6. Lineage-Specific HSPG Methylation Could Be Driving ReNcell Signaling Pathway Differences

**Figure 7:**
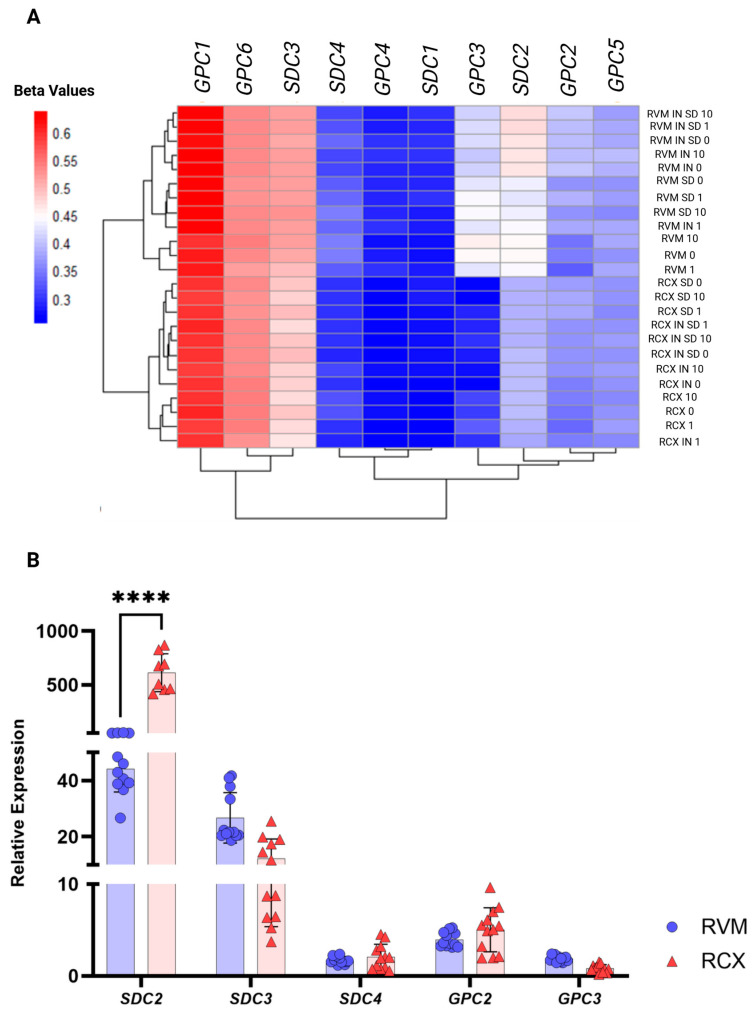
**Distinct HSPG methylation across ReNcell cultures.** (**A**) Average methylation values were assessed in ReNcell CX (RCX) and VM (RVM) cultures, revealing distinct methylation patterns in *SDC2*, *SDC3*, *SDC4*, and *GPC3* as the most differentially methylated HSPGs between the two cell types. In all cases, higher methylation levels were observed in RVM cultures. For *SDC3*, the primary differences were attributed to specific CpG sites located within the 200 bp downstream region of its transcription start site. Only the heparin-treated monolayer samples are shown in the accompanying table, highlighting beta values from treatments with 1 µg/mL and 10 µg/mL heparin. Beta values reflect the proportion of methylated cells at a given locus (e.g., 0.2 indicates 20% methylation). (**B**) Relative gene expression levels were quantified using the ∆∆Ct method and scaled by 10^6^ for visualisation. Statistical significance is indicated by **** *p* < 0.0001.

Clear differences in methylation were identified for the HSPG core protein genes *SDC2*, *SDC3*, *SDC4*, *GPC2,* and *GPC3* (Figure 7). In particular, a relationship between methylation at the *SDC2* CpG site *SDC2*:cg26777303 along with changes in gene expression was identified in ReNcell cultures consistent with previously published data in hNSC-H9 cells [13]. In RCX monolayer cultures, this site was found to be associated with a beta value of 0.651, with relative gene expression measured at 347.3 ± 43.1. In contrast, RVM monolayers exhibited higher methylation at this site (beta value = 0.762) along with significantly lower gene expression (32.6 ± 2.3), indicating potential gene expression regulation through *SDC2*:cg26777303. Assessment of *SDC3* identified lower methylation observed in RCX when compared to RVM cultures, particularly at 200 bps downstream of the TSS (TSS200) region of the gene. A consistent decrease in methylation of over 10% was also observed following augmentation of the cultures with 1 µg/mL of heparin, which was also found to be associated with a significant increase in gene expression from 12.3 ± 6.9 (untreated) to 23.8 ± 10.2 (*p* = 0.0019). All sites within *SDC4* available on the arrays were found to be hypomethylated in both the RCX and RVM monolayer cultures, with the exception of *SDC4*:cg18622281, located closest to the TSS. *GPC2* methylation showed modest responses to heparin treatment and *GPC3* methylation was found to be low in all RCX cultures. In the RVM cultures, methylation differences were found within a CpG island (chrX:133118960–133119990) overlapping the TSS and the 1st Exon. However, no corresponding changes in gene expression were detected, suggesting that methylation at this locus may not directly influence *GPC3* transcription.

### 3.7. SDC4 Is Necessary for Cell Type-Specific Notch Activation

**Figure 8:**
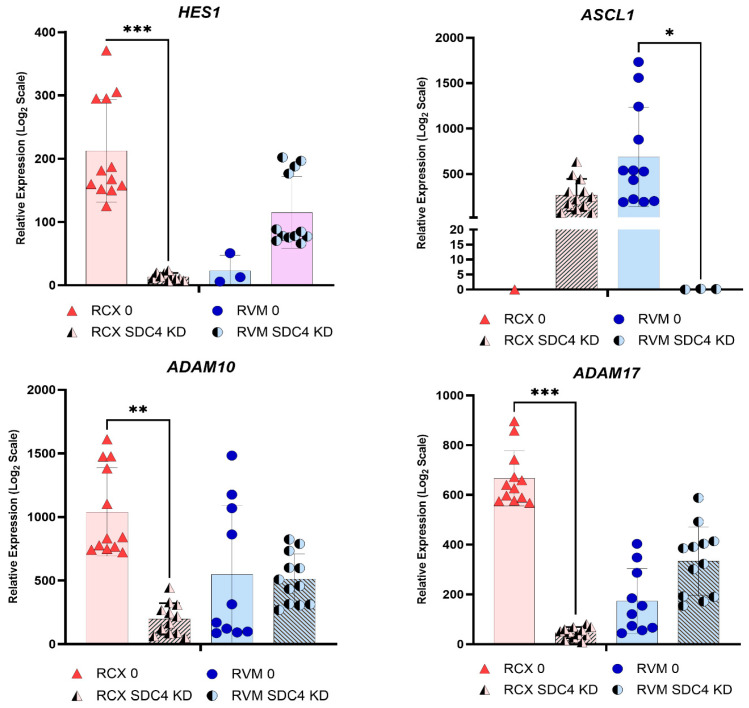
**Gene expression of Notch pathway genes in *SDC4* knockdown (KD) ReNcell cultures.** ReNcell CX (RCX) and VM (RVM) cultures were maintained under growth factor-free conditions to model early spontaneous differentiation (neuroinduction). *SDC4* were depleted using siRNA, and samples were collected 72 h post-KD. Relative gene expression was quantified using the 2^(−ΔCt)^ method and scaled by 1 × 10^6^ to facilitate visualisation. Statistical significance is indicated with asterisks: * *p* < 0.05, ** *p* < 0.001, *** *p* < 0.0005. Changes in expression of canonical Notch targets (*HES1*, *ASCL1*) and Notch-related enzymes (*ADAM10*, *ADAM17*) highlight differential modulation of the Notch signaling pathway between RCX and RVM models in response to SDC4 KD.

To further interrogate the role of HSPGs in Notch-related signaling, *SDC4* was selected for targeted functional investigation. This decision was informed by the observation that *SDC4* gene expression exhibited oscillatory dynamics in both RCX and RVM models that closely paralleled those of the canonical Notch target gene *HES1* (Supplementary Figure S2). Given that *HES1* expression is a hallmark of active Notch signaling during neurogenesis, the concordant temporal expression pattern of *SDC4* suggested a potential regulatory association with Notch pathway activity. siRNA knockdown (KD) experiments in ReNcell models under growth factor-deprived neuroinductive conditions (absence of EGF/FGF) revealed distinct interactions between *SDC4* and the Notch signaling pathway (Figure 8). As previously observed, RCX cultures maintained high basal Notch signaling, whereas RVM cultures did not. In RCXs, early differentiation following *SDC4* KD led to decreased *HES1* expression, a canonical Notch target, accompanied by increased *ASCL1*, indicating that *SDC4* depletion may inhibit Notch signaling and promote neuronal differentiation in RCX cultures. In contrast, RVM exhibited increased *HES1* and decreased *ASCL1*. Corresponding changes in Notch receptor expression were also observed: RCX displayed decreased *NOTCH2* alongside elevated *ASCL1*, whereas RVM showed decreased *NOTCH1* with elevated *HES1* under SDC4 KD. Additionally, expression of *ADAM10* and *ADAM17* was found to be markedly reduced in RCX but slightly increased in RVMs, further supporting a differential modulation of Notch signaling by *SDC4* between the two cell models.

## 4. Discussion

The primary aim of this study was to define DNA methylation patterns that distinguish neural lineage potential in human NPC lines, with particular emphasis on astrocytic/glial versus neuronal trajectories. A broader objective was to assess whether commonly used ReNcell models harbour intrinsic lineage biases characterised by differences within their DNA methylation landscape. Furthermore, whether this influenced cell lineage differentiation capacity and suitability for neurogenesis research, potentially paving the way for more refined and shorter *in vitro* protocols to direct lineage specification. Together, our findings highlight clear and biologically meaningful DNA methylation differences between the RCX and RVM cultures, revealing that these models occupy distinct positions along the neurogenic lineage continuum.

These lineage tendencies were supported by global methylation of distinct neural marker genes. RCX cultures displayed pronounced hypomethylation of *DCX* and *ENO2* -two genes tightly linked to immature neuronal stages [35,36]. In contrast, *GFAP* was found to be consistently hypomethylated in RVM cultures. Although *GFAP* is classically considered an astrocytic lineage marker, its presence in early multipotent progenitors suggested that RVM cells may occupy a glial-primed or less lineage-restricted progenitor state [37]. Intriguingly, RVM cultures diverged most strongly from defined neural lineages under neurosphere (IN SD) conditions, consistent with a shift toward a more multipotent state. RCX samples, in contrast, were observed to remain more stable and clustered closer to each other and to neuronal-associated signatures under basal or SD culture conditions. These findings suggest RCX cultures reside further along neuronal commitment, whereas RVM cultures require inductive cues to transiently approximate RCX-like states.

Collectively, these methylation signatures indicate that RCX cultures are more developmentally advanced and perhaps neuronal-biased, whereas RVM cultures remain more glial-oriented and developmentally immature. Notably, although both ReNcell models showed greater similarity to primary glial methylation profiles, this finding must be contextualised by a central limitation: the inherent mismatch between immortalised, foetal-derived NPCs and fully differentiated primary neural subtypes. Differences in developmental stage, immortalisation-induced epigenomic remodelling, tissue origin, and culture-level heterogeneity can profoundly shift methylation landscapes, inevitably influencing correlation outcomes. Nevertheless, even within this framework, the analysis serves as a valuable pilot reference point, highlighting consistent lineage-relevant trends and revealing key mechanistic directions for future work.

Furthermore, the HSPG *SDC2* was confirmed to be hypomethylated and more highly expressed in RCX cultures, in line with its established role—along with *SDC3*—in neuronal lineage commitment [38]. Conversely, *SDC4*, often associated with oligodendrocytic and glial traits, exhibited uniformly low methylation across both models with only minor inter-culture differences. Notably, *SDC2*, *SDC3*, and *GPC3* were all found to be hypomethylated in RCX cells, suggesting that the “epigenetic brake” for these HSPGs is released in this cell model, rendering them more transcriptionally permissive for neuronal differentiation cues.

The divergent methylation of RCX and RVM are likely rooted in their cellular origins. RVM cells derive from heterogeneous foetal brain tissue and therefore represent a mixed NPC population. RCX cells, in contrast, originate from a monoclonal line, capturing a single cell type—likely—at a more advanced stage of lineage commitment [23]. This intrinsic difference may explain why RCX cultures show relatively minor methylation changes across culture conditions, whereas both models—particularly RVM—undergo substantial shifts when transitioning from 2D to 2.5D (IN) formats. IN conditions have previously been shown to reinforce multipotent NPC states [39,40,41], consistent with our observation that IN cultures drive both RCX and RVM away from defined neural subtype signatures.

Notch signaling further underscores these lineage distinctions. RVM cultures exhibited elevated *DLL1* and *ASCL1* expression, suggesting low basal Notch pathway activity and a reliance on lateral inhibition [42]. *DLL1*-mediated lateral inhibition is typically associated with heterogeneity, as *DLL1*-expressing cells restrict neighbouring cells from differentiating—consistent with the mixed progenitor character of RVM cultures [43]. RCX cultures, in contrast, displayed higher *JAG* ligand expression, associated with lateral induction, a mechanism that promotes coordinated differentiation and yields more homogeneous cultures [44,45]. These ligand-specific differences imply that RCX cultures maintain an active Notch environment conducive to synchronous lineage progression, whereas RVM cultures maintain a heterogeneous, less synchronised progenitor pool. The presence of *DLL3*, a known inhibitory ligand [46], further suggests fine-tuning of Notch responsiveness in both models. The decline of *DLL3* in RVM cultures by D7, accompanied by increased *NOTCH1*, *NOTCH2,* and *DLL1*, hints at a dynamic regulatory environment in which ligand composition shifts with developmental stage and extracellular context (e.g., laminin vs. Geltrex, availability of HSPGs).

Active Notch signaling is crucial for maintaining NPC self-renewal and suppressing premature neurogenesis [3]. The marked reduction in Notch gene expression at D7 in RCX cultures supports the view that RCX cells are intrinsically further along the neurogenic trajectory than RVM cells. Conversely, VM IN cultures showed downregulation across all Notch pathway enzymes, reinforcing the notion that IN conditions stabilise a multipotent, less lineage-restricted state [47]. Importantly, CX IN cultures retained detectable Notch activity, further emphasising their relatively advanced developmental status when compared to the more immature RVM phenotype.

The functional consequences of these intrinsic differences were evident in the *SDC4* KD experiments. RCX cultures, which predominantly rely on *NOTCH2–JAG* interactions, displayed reduced Notch activity following reduced *SDC4*—consistent with decreased *HES1* and increased *ASCL1* expression. This suggested that *SDC4* may preferentially scaffold *NOTCH2*-containing complexes in these cells. In contrast, RVM cultures appeared to compensate via increased *NOTCH1* signaling, reflected by elevated *HES1* expression following *SDC4* KD. This divergent response highlights fundamental differences in receptor–ligand usage, co-factor availability, and baseline Notch tone between the two models.

Several limitations of this study should be acknowledged. First, RCX and RVM models were grown utilising distinct attachment factors, which may have contributed to differences in HSPG expression profiles independent of intrinsic cellular regulation. To mitigate this, analyses were extended to include IN cultures, which are not dependent on substrate attachment, thereby partially reducing any potential culture-related confounding effects. Second, CpG methylation changes identified in this study were not independently validated using targeted approaches such as bisulfite sequencing [48]. Consequently, the findings should be interpreted as hypothesis-generating rather than definitive evidence of functional epigenetic regulation. Nonetheless, the Illumina Infinium MethylationEPIC array has been widely validated as a highly reproducible platform providing a robust foundation for the identification of biologically relevant methylation patterns that warrant further investigation [49]. Third, associations were explored between DNA methylation and gene expression. These relationships remain inferential, as protein-level validation was not performed. As a result, conclusions regarding downstream functional consequences and signaling pathway activity should be interpreted with appropriate caution.

Future studies should aim to validate methylation changes using targeted bisulfite-based assays and extend molecular characterisation of the cultures to the protein level. This could include immunodetection of key signaling components (e.g., Notch ligands and receptors) to confirm proposed regulatory interactions and strengthen mechanistic inferences. While this study focused on functional interrogation of *SDC4*, inherent DNA methylation differences observed in *SDC2* and *GPC3* suggest that these HSPGs may also play important, yet distinct, regulatory roles and warrant targeted functional investigation in future studies. In addition, in some cases, this study identified areas of high CpG DNA methylation that were not accompanied by corresponding reductions in gene expression. This discordance highlights the complexity of epigenetic regulation, suggesting methylation at specific loci may exert context-dependent or non-canonical regulatory functions beyond simple transcriptional repression.

Collectively, these observations raise important questions regarding the functional relevance of site-specific DNA methylation, its relationship to chromatin context and transcriptional regulation, and its contribution to signaling pathway modulation during neurogenesis. Addressing these questions through targeted epigenetic and functional assays represents a key direction for future work

## 5. Conclusions

Taken together, this data provides a coherent mechanistic framework for understanding the divergent lineage behaviours of RCX and RVM cultures. RCX cells represent a more homogeneous and more mature NPC population characterised by active *JAG–NOTCH2* signaling and permissive methylation of neuronal and HSPG-associated genes. RVM cells constitute a more heterogeneous, glial-oriented progenitor pool with a Notch landscape shaped by *DLL1*-mediated lateral inhibition and developmentally immature epigenetic features. These distinctions have important implications for experimental design and highlight the need to consider intrinsic lineage biases when selecting ReNcell and other models for neurodevelopmental or neurodegenerative research.

## Figures and Tables

**Table 1:** **Summary of enriched gene ontology (GO) networks in ReNcell CX (RCX) and ReNcell VM (RVM) cell models based on differentially methylated genes.** Gene ontology networks were identified through STRING (v12.0) enrichment analysis using genes that exhibited differential methylation at a minimum of two CpG sites within the top 1000 most variable loci. GO terms shown reflect biological processes significantly enriched within each cell model. The “Count in network” indicates the number of genes from the input list mapping to each GO term. “Strength” refers to the magnitude of enrichment, calculated as the log ratio of observed versus expected gene counts, where higher values reflect stronger enrichment. The false discovery rate (FDR) is reported as an adjusted *p*-value, indicating the estimated proportion of false positives; lower FDR values represent higher statistical confidence. GO pathways were sorted based on strength (e.g., most likely involved). Together, these data highlight distinct biological processes associated with lineage-specific methylation patterns in RCX and RVM models.

**Enriched in RCXs**				
**Gene** **Ontology Term**	**Description**	**Count in** **Network**	**Strength**	**False** **Discovery Rate**
GO:0072133	Metanephric Mesenchyme morphogenesis	2 of 3	2.43	0.0285
GO:0021902	Commitment of neuronal cell to specific neuron type in forebrain	2 of 6	2.13	0.0477
GO:0097154	GABAergic neuron differentiation	3 of 16	1.88	0.0112
GO:0021983	Pituitary gland development	4 of 43	1.57	0.0058
GO:0009954	Proximal/Distal pattern formation	3 of 35	1.54	0.0397
GO:0045746	Negative regulation of Notch signaling pathway	3 of 38	1.5	0.0454
**Enriched in RVMs**				
**Gene** **Ontology Term**	**Description**	**Count in Network**	**Strength**	**False** **Discovery Rate**
GO:0217787	Oligodendrocyte cell fate specification	2 of 4	2.43	0.0195
GO:0021781	Glial Cell Fate Commitment	4 of 12	2.25	0.00029
GO:0021796	Cerebral cortex regionalisation	2 of 7	2.18	0.0342

## Data Availability

The original contributions presented in this study are included in the article/Supplementary Material. Further inquiries can be directed to the corresponding author.

## Supplementary Materials

The following supporting information can be downloaded at: https://www.mdpi.com/article/10.3390/biology15030231/s1, Table S1: CpG sites with the strongest positive and negative loadings for each principal component identified by principal component analysis, indicating methylation features underlying the positioning of RCX and RVM cells relative to primary neural cell types., Figure S1: Hierarchical clustering of methylation patterns in (A) TGFβ and (B) Wnt signaling pathway genes across RCX and RVM cultures. Panels display beta values representing DNA methylation levels, with blue indicating low methylation (potentially permissive for gene expression) and red indicating high methylation (potentially repressive). Each value reflects the average methylation across all CpG sites associated with a given gene. Notably, panel A shows a maximum beta value of 0.45, indicating intermediate methylation levels overall, even in comparison to panel B. For reference, a beta value of 0.3 suggests ~30% methylation at that locus. Figure S2: Gene expression profiles of syndecans (SDC1–4) were examined along with key downstream targets of Notch signaling (ASCL1 and HES1) across the ReNcell CX (red) and ReNcell VM (blue) models. Among the syndecan family members, SDC4 displayed expression dynamics that most closely paralleled those of HES1 in both cell lines, with broadly concordant temporal trends observed across differentiation time points. In contrast, SDC1 exhibited a similar expression pattern to HES1 in ReNcell VM cells (blue) but not in ReNcell CX cells (red), while SDC3 showed partial similarity to gene expression profiles in ReNcell CX cells but not in ReNcell VM cells. Although the observed associations between SDC4 and HES1 were indirect and trend-based, SDC4 was the only syndecan demonstrating consistent behaviour across both models. This cross-model concordance formed the primary rationale for prioritising SDC4 for subsequent analyses.

## Abbreviations

The following abbreviations are used in this manuscript:

NPC	Neural progenitor cell
RVM	ReNcell VM (ventral mesencephalon derived)
RCX	ReNcell CX (cortex derived)
DCX	Doublecortin
ENO2	Enolase 2
MAP2	Microtubule Associated Protein 2
GFAP	Glial fibrillary acidic protein
TGFb	Transforming Growth Factor Beta
JAG1-2	Jagged 1-2
DLL1-3	Delta like 1-3
SDC4	Syndecan 4
NSC	Neural stem cell
AD	Alzheimer’s Disease
HSPG	Heparan sulfate proteoglycan
FGF	Fibroblast growth factor
SD	Neuro-inductive (or spontaneously differentiated)
IN	Induced neurosphere
O2-A	Oligodendrocyte-type 2 astrocyte
PBS	Phosphate buffered saline
DMEM	Dulbecco’s modified eagle medium
DMSO	Dimethylsulfoxide
EGF	Epidermal growth factor
POLA1	DNA polymerase alpha catalytic subunit
SLC9A1	Sodium/hydrogen exchanger 1
FOXA1/B1/P2	Forkhead box A1/B1/P2
GATA2	GATA Binding Protein 2
PITX1	Paired Like Homeodomain 1
DLX1	Distal-less homeobox 1
PCA	Principal Component Analysis
TMEM175	Transmembrane protein 175
KPNA1	Karyopherin subunit alpha 1
SEC14L1	SEC14-like lipid binding 1
NES	Nestin
TUBB3	Tubulin beta 3
S100B	S100 calcium binding protein
nES	Normalized enrichment score
MIR9-2HG	MIR9-2 Host Gene
NDUFS5	NADH:Ubiquinone Oxidoreductase Subunit S5
TBR1	T-box brain transcription factor 1
SATB2	Special AT-rich sequence-binding protein 2
NEUROD1	Neuronal differentiation 1
SOX9	SRY-box transcription factor 9
GLI3	GLI family zinc finger 3
FZD1	Frizzled class receptor 1
PROX1	Prospero homeobox 1
NR2F2	Nuclear receptor subfamily 2 group F member 2
NKX6-1	NK6 homeobox 1
WNT5A/B	Wnt family member 5A/B
NRG1	Neuregulin 1
KD2MB	Lysine Demethylase 2B
NeuN	Neuronal Nuclei
GNAS-AS	GNAS Antisense RNA
EYA4	EYA Transcriptional Coactivator and Phosphatase 4
MIR124-2	MicroRNA 124-2
SLC4A2	Solute Carrier Family 4 Member 2
LIMK1	LIM domain kinase 1
ZIC1	Zinc Family Member 1
F2RL1	F2R Like Trypsin Receptor 1
SIAH3	Siah E3 Ubiquitin Protein Ligase 3
SNED1	Sushi, Nidogen and EGF Like Domains 1
ADAM10-17	A disintegrin and metalloproteinase domain-containing proteins 10–17
PSEN1-2	Presenilin 1–2
TSS	Transcription starting site
HES1	Hes family bHLH transcription factor 1
ASCL1	Achaete-scute family bHLH transcription factor 1
KD	Knockdown
SCR	Scramble
UT	Untreated
