# Supplementary figures and images for: DNA Methylation Landscape of ReNcell Common Neural Progenitor Cell Lines Reveals Distinct Lineage Bias
Source: Biology (Basel). 2026 Jan 26;15(3):231. doi: 10.3390/biology15030231 (PMC12897062; doi:10.3390/biology15030231)

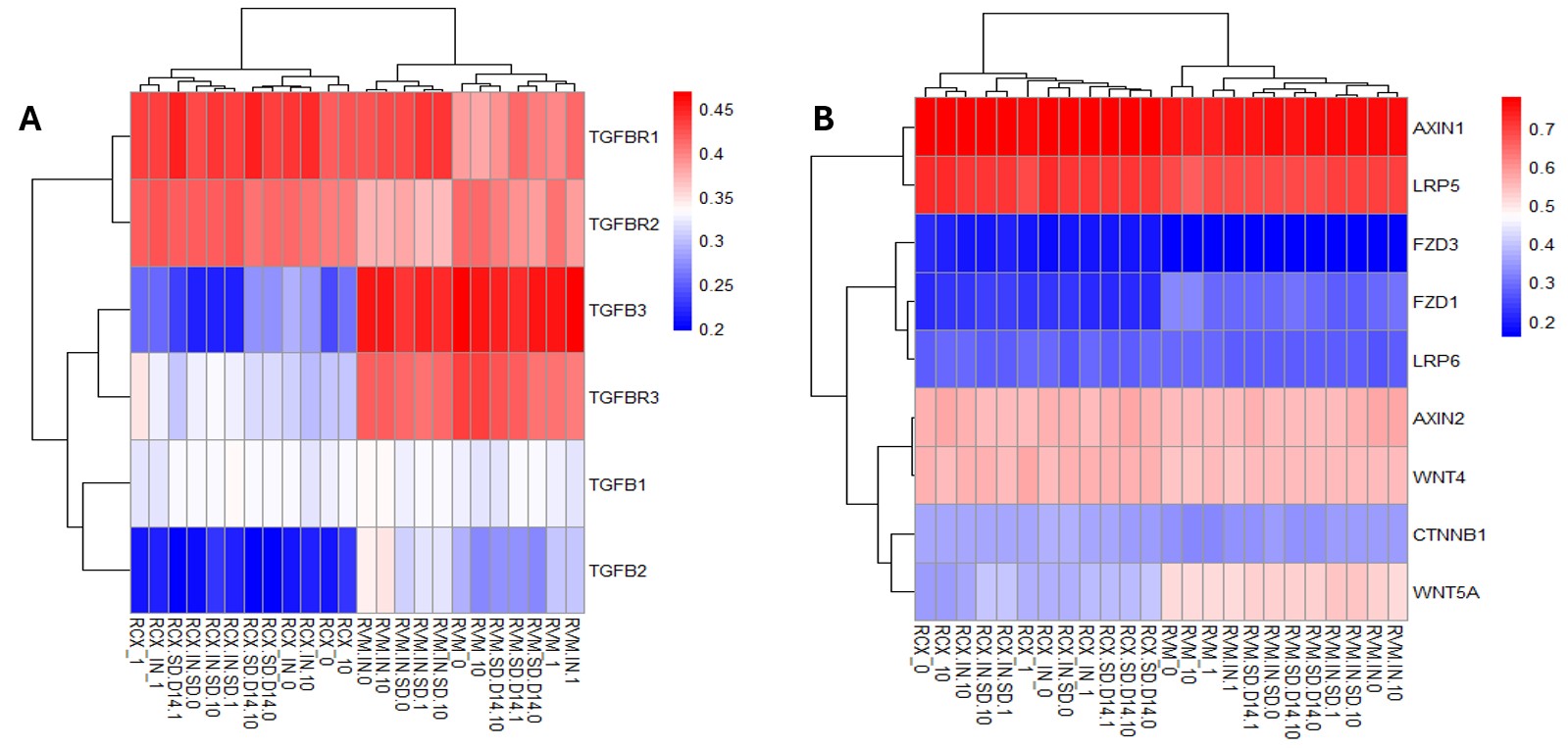

Supplement: Supplementary file 1 [file biology-15-00231-s001.zip › Figure S1.jpg]

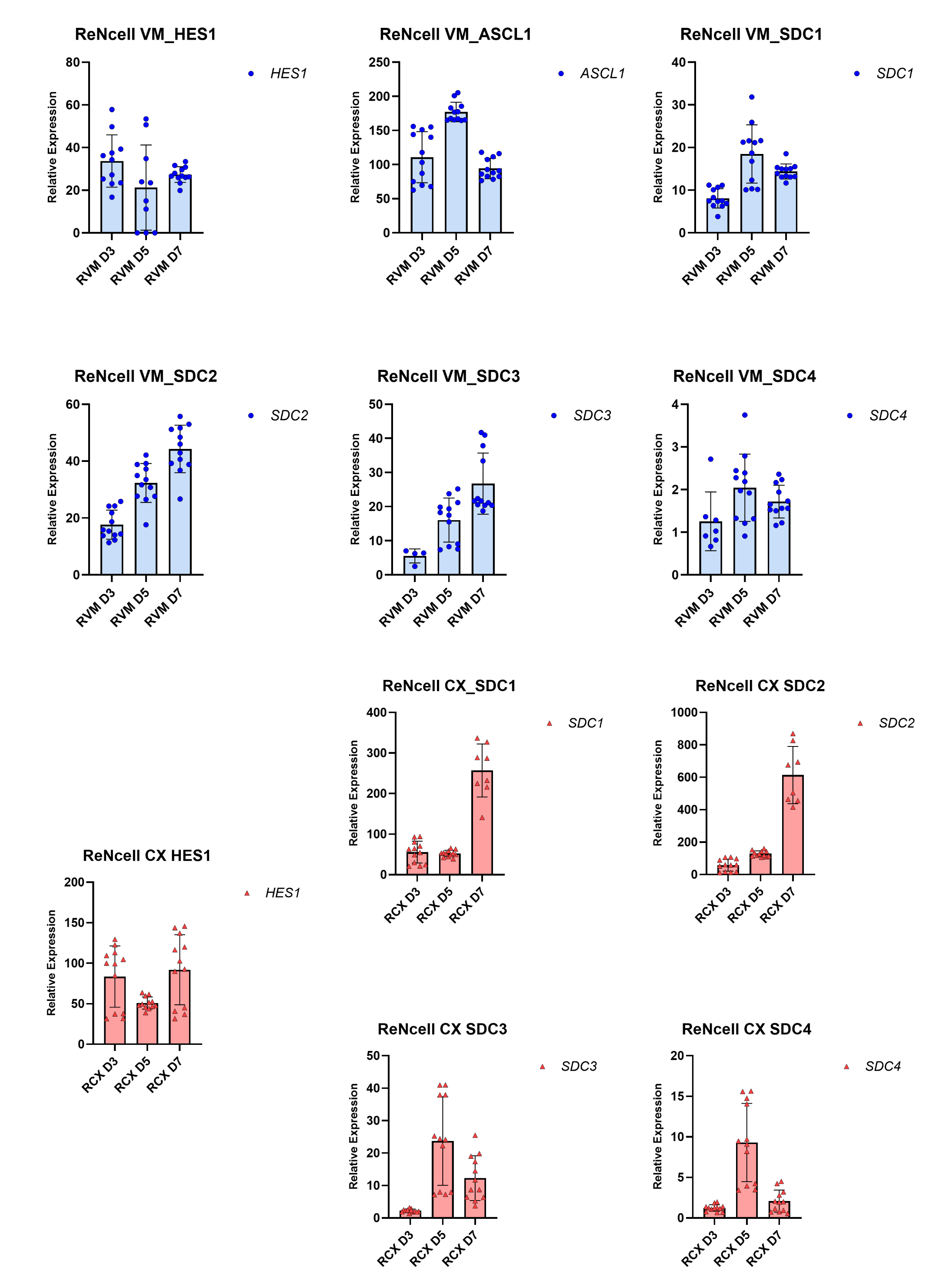

Supplement: Supplementary file 1 [file biology-15-00231-s001.zip › Supplementary S2.png]
